# Depression signs in South Asian medical students studying abroad

**DOI:** 10.1192/j.eurpsy.2026.11639

**Published:** 2026-07-31

**Authors:** O. V. Nikolaeva, E. N. Tashkenbaeva, J. N. Daminov, T. E. Nikolaeva

**Affiliations:** 1 Department of Hospital Therapy, I.N. Ulianov Chuvash State University, Cheboksary, Russian Federation; 2 Department of Internal Medicine and Cardiology No. 2; 3 Faculty of Pharmacy, Samarkand State Medical University, Samarkand, Uzbekistan; 4 Faculty of Medicine, I.N. Ulianov Chuvash State University, Cheboksary, Russian Federation

## Abstract

**Introduction:**

Depression is a frequent mental health problem for medical students, especially those who study abroad. How does studying in culturally similar or different countries influence the depression signs in South Asian students?

**Objectives:**

Our objective was to compare the signs and intensity of depression in South Asian medical students studying in Uzbekistan and Russia.

**Methods:**

With the help of the Beck Depression Inventory (BDI-II), we surveyed anonymously 116 Indian and Pakistani medical students studying in Uzbekistan and Russia. The surveyed group included 62.93% of men and 37.07% of women, with the mean age – 22.90±2.59. Groups of students in Uzbekistan (50 people) and Russia (66 people) did not statistically differ in age and gender (p>.05).

**Results:**

The mean level of depression, as well as cognitive-affective and somatic-affective symptoms of 116 respondents exceeded the norm (p<.0001) more than one and a half times. At the same time, over a half of the students (53.45%) showed none or minimal signs of depression. Every tenth student revealed signs of mild depression (10.34%), every seventh had moderate depression (14.66%), and every fifth suffered from severe depression (21.55%). The analysis of the indicators in each country did not reveal any reliable difference. Nevertheless, we found certain peculiarities in the specificity of depression symptoms manifestation. Thus, South Asian students in Russia showed maximally expressed changes in appetite (1.00±0.89) and self-criticism (0.98±1.06), those in Uzbekistan revealed loss of pleasure (1.08±1.05) and guilty feelings (1.04±1.12). The third manifested symptom in both groups was the same – changes in the sleep pattern (1.02±0.84 in Uzbekistan and 0.97±0.78 in Russia).

**Conclusions:**

Signs of depression are equally prevalent in half of the South Asian medical students studying in both Uzbekistan and Russia. The similarity of their manifestation may be due to an objective complexity of medical education, the difference – due to the cultural specificity of the country where they study. Mental health professionals might apply the received findings with a view to taking care of the international students’ health and well-being.

**Disclosure of Interest:**

None Declared

